# Systematic lupus erythematous patients following COVID-19 vaccination: Its flares up and precautions

**DOI:** 10.1016/j.amsu.2022.104282

**Published:** 2022-07-31

**Authors:** Govinda Khatri, Priya, Somina Shaikh, Aashish, Aneesh Rai, Huzaifa Ahmad Cheema, Mohammad Yasir Essar

**Affiliations:** a Department of Internal Medicine, Dow Medical College, Dow University of Health Sciences, Karachi, Pakistan; b Department of Medicine, King Edward Medical University, Lahore, Pakistan; c Kabul University of Medical Sciences, Kabul, Afghanistan

**Keywords:** Systemic lupus erythematosus, Lupus flare-up, COVID-19 vaccine, mRNA vaccine, Autoimmune adverse effects

## Abstract

Systemic lupus erythematosus (SLE) is an autoimmune disease that can cause both direct and indirect inflammatory damage to multiple organs. Clinical symptoms in the skin, joints, kidneys, and central nervous system, as well as serological indicators such as antinuclear antibodies (ANA), notable antibodies to dsDNA, are used to diagnose SLE. mRNA SARS-CoV-2 vaccines have been shown to trigger SLE flares and the development of new rheumatic diseases. SARS-CoV-2 mRNA vaccinations increase type I interferon (INF), which is not only known to have a role in the antiviral response but is also a crucial cytokine in the pathophysiology of SLE. Furthermore, both the mRNA and adenovirus vaccines boost the production of type 1 interferons, which are required for the spread of SARS-CoV-2. The danger of not administering the COVID-19 vaccination to SLE patients is significantly larger than the likelihood of its adverse effects, which are most likely caused by intrinsic immune failure, demographic disease activity, medications, linked organ damage, and comorbidities. The adverse effects of COVID-19 vaccination in SLE patients are common (about 50%), although they do not interfere with daily functioning in the majority of cases. Several precautions can be taken to avoid the complications associated with COVID-19 vaccinations.

## 1 Introduction

Systemic lupus erythematosus (SLE) is an autoimmune disease characterized by the presence of antinuclear antibodies (ANA) and the production of immunological complexes, which can cause inflammatory damage to many organs both directly and indirectly [1]. While the frequency of SLE is higher among reproductive-age, nonwhite women, the disease can show at any age, gender, or ethnic background. The interplay of genetic and environmental variables, including UV light exposure, cigarette smoking, and infections, plays a role in its pathogenesis [2]. SLE is diagnosed based on clinical symptoms in the skin, joints, kidneys, and central nervous system, as well as serological markers such as antinuclear antibodies (ANA), particularly antibodies to dsDNA. Patients with SLE have reduced amounts of cytotoxic T and CD4 T lymphocytes, both of which are important modulators of the humoral immune response [3].

Coronavirus disease-19 (COVID-19), caused by the severe acute respiratory syndrome coronavirus (SARS-CoV-2) virus is declared a global emergency by World Health Organization (WHO), which has several socioeconomic impacts globally [4,5]. Many preventative approaches and non-pharmaceutical treatments have been used to decrease disease spread, including infection control, patient isolation, and social distancing. Continues to be a global threat [6]. Vaccination is one of the most effective therapies for overcoming it [7]. Several vaccinations, such as hepatitis B, human papillomavirus, and measles, have been associated with SLE. Recently, it has been observed that mRNA SARS-CoV-2 vaccinations may cause SLE flares and the development of new rheumatic illnesses [8]. Recent research on SLE patients found a mild to moderate illness flare in 11.4% of patients and a severe flare in 1.3% of patients following COVID-19 vaccination [9]. Two instances of new-onset SLE following SARS-CoV-2 mRNA immunization have been described, both with cutaneous symptoms [10]. Kreuter et al. described a 79-year-old man who had lethargy as well as papulosquamous and annular skin eruptions ten days after receiving the BNT162b2mRNA vaccination [11]. The hypothesized mechanism might be that an inactive viral component or attenuated microbe generates molecular mimicry or bystander activation in genetically susceptible people [12]. Adjuvants, which are used to boost immunity in certain vaccinations, can also cause autoimmune reactions. Given the temporal association with COVID-19 vaccination and the biological plausibility of the vaccines inducing autoimmune reactions, COVID-19 vaccination may be a probable trigger for the progression or development of SLE. Hence, this review aims to explore the available literature regarding the association of SLE with COVID-19 vaccination, the possible underlying mechanism and the precaution techniques that can be adopted.

### 1.1 Mechanism of COVID-19 vaccine-induced SLE

Vaccinations are widely regarded as a safe and efficient method of preventing serious viral diseases and limiting infection spread in patients with autoimmune diseases. Three COVID-19 vaccines have been approved for emergency use: mRNA vaccines (Pfizer and Moderna) and viral vector vaccines (Johnson & Johnson (J&J)) [13]. At least one dose of the COVID-19 vaccine has been administered to 65% of the world's population. Globally, 11.45 billion dosages have been administered, with 11.79 million doses being administered daily [14]. The first month of vaccine observation showed that 90.9% of people experienced non-serious adverse events, such as local or systemic reactions [15].

A few astonishing cases were presented which showed signs of SLE following COVID-19 vaccination. Adverse responses to various types of vaccinations may develop as a result of interactions between the host's susceptibility and vaccine components. One of the processes implicated in such responses is molecular mimicry [16].

SLE's precise aetiology is likely complex and currently, it is only partially understood. The clinical symptoms of SLE such as nephritis, pleuritis, cardiomyopathy, and dermatitis are mediated by antibody production and the development of immune complexes, resulting in small-vessel vasculitis. B-cell hyperactivity and increased autoantibody production have been implicated in the pathogenesis of SLE, according to research [17]. Furthermore, the activation of CD4^+^ immune pathways has been shown to have a role in the pathogenesis of SLE [18].

Kanduc et al. examined peptide sharing between the SARS-CoV-2 spike protein and human proteins and discovered significant sharing at the heptapeptide level [19] suggesting that antibodies produced in response to the vaccine may cross-react with the host and cause autoimmune diseases in susceptible individuals [20]. Following mRNA vaccination, various pro-inflammatory pathways are triggered as a result of the mRNA itself, liposomes or lipid nanoparticles used to encapsulate the mRNA, and receptor binding essential for spike protein translation [21]. Because mRNA is a single-stranded RNA, it is a toll-like receptor (TLR-7) ligand [22]. TLR-7 expression is higher in SLE humans and mice [23,24]. Furthermore, both the mRNA and adenovirus vaccines increase the synthesis of type 1 interferons, which are essential for the establishment of SARS-CoV-2 immunity [25] but have also been linked to the pathogenesis of SLE and other autoimmune diseases [26,27]. Increased amounts of Th1 cells, as well as Th1-associated cytokines and chemokines, have been observed in skin lesions in cutaneous lupus erythematosus (CLE) [28]. TNF-alpha and IFN-gamma have a function in CLE pathogenesis by promoting cytokine release and immune cell recruitment [29]. In autoimmune diseases like SLE patients are usually on immunosuppressants which enhances the chances of serious infection following COVID-19 infection. However, the risk of not administrating the COVID-19 vaccine in SLE patients is far greater than its adverse effects. The ACR presently recommends that people with autoimmune and inflammatory rheumatic illness, including lupus, get multi-dose mRNA vaccinations (Pfizer or Moderna) if they are available, rather than the single dosage vaccine (Johnson & Johnson). At least two Pfizer or Moderna vaccines are included in the series. The ACR and CDC further suggest that persons with lupus who are moderately to severely immunocompromised get a third dose of an mRNA vaccine at least 28 days after the first two immunizations are completed. This implies that persons who have had two injections of the Pfizer or Moderna mRNA vaccine and are immunocompromised should have a third shot, but it is no longer referred to as a booster; instead, it is included in the original vaccination series recommended [30].

### 1.2 Effect of COVID-19 vaccine in SLE patients

Several studies have found that people with SLE are more likely to contract SARS-CoV-2 and have worse outcomes from COVID-19, most likely due to intrinsic immune failure, demographic disease activity, medicines, related organ damage, and comorbidities [31]. As a result, individuals with autoimmune diseases such as SLE have been selected as the first to get the COVID-19 vaccination due to their susceptible symptoms. However, because of concerns about adverse effects and a lack of long-term data on vaccine safety, a high number of individuals with autoimmune diseases show vaccination rejection or ambivalence [32]. In addition to the COVID-19 vaccination, individuals with SLE have reported a poor immune response to vaccines against other pathogens such as influenza and pneumococcal vaccine [33]. Tang W et al. discussed different studies on the effects of the COVID-19 vaccine in autoimmune disorders patients in their research article, and they concluded that immunosuppressive treatments, rather than the underpinning autoimmune disease, might be the foremost virulence factor restricting immune responses against SARS-CoV-2 vaccines, and these immunosuppressive treatments were found to be a significant risk factor for diminished immunogenicity [34]. Glucocorticoids, methotrexate, mycophenolate or mycophenolic acid, and rituximab are examples of immunosuppressive therapies.

Both SLE and COVID-19 pathophysiology have been linked to the activation of comparable molecular structures, such as type 1 interferon and proinflammatory cytokine pathways [35]. COVID-19 infection causes the release of autoantibodies in the serum of hospitalized patients, resulting in severe autoimmunity in these individuals that is exacerbated by COVID19 infection. SLE patients infected with COVID-19 have multiple inflammatory flare-ups, including arthritis, alopecia, rash, pleurisy, and serological deterioration (poorly competent C3/C4 components and antibodies to double-stranded DNA) [35]. The most frequent side effects of vaccination include soreness at the injection site, weariness, and headache. In individuals with autoimmune illnesses such as SLE, however, immunization causes more severe symptoms such as tiredness, fever, headache, chills, muscular discomfort, and joint pain [36]. According to the international vaccination against COVID in systemic lupus (VACOLUP) initiative, COVID-19 immunization was well accepted in SLE patients, despite the risk of flares. The negative effects of COVID-19 immunization in SLE patients are prevalent (approximately 50%), although they do not interfere with everyday functioning in the majority of instances [37].

### 1.3 Complications and disease flare-up risk following vaccination

A case report showed the development of Evans syndrome associated with SLE and worsening of bronchial asthma after administration of mRNA COVID-19 vaccine [38]. In another study, patients with rheumatic disorders reported joint problems more frequently than controls (49 [10%] vs 3 [1%]), although only a small percentage of patients (26 [5%]) reported a worsening of their autoimmune condition up to 2 months following COVID-19 immunization [39]. Watad et al. evaluated immune-mediated disease (IMD) flares or fresh autoimmune disease onset after COVID-19 vaccination in five post-secondary centres located in various countries with initial vaccination adoption. Only 10 of the 27 individuals included in the study had a fresh beginning of IMD, and all of them had any form of IMD-related symptoms. None of the ten individuals had a new onset of SLE [40]. A case report illustrated the relapse of SLE in a 42-year-old lady following SARS-COV-2 vaccination. It's important to remember that the COVID-19 mRNA vaccination might cause a recurrence of immune-mediated disease [41]. According to the findings of a recent study, COVID-19 immunization appears to be well tolerated in SLE patients, with just a little likelihood of flare, if any, even after mRNA vaccination [42].

### 1.4 Precautionary measures

Table 1 outlines some precautionary strategies for reducing the risk of SLE caused by COVID-19 vaccination.

**Table 1:** Precautionary measures for control of COVID-19 vaccination-induced SLE.

Precautionary measures	
**1.**	Continue standard treatment to avoid a disease flare of SLE that worsens COVID-19 complications & necessitates the use of corticosteroids [43].
**2.**	Take care that: • Patients with recently diagnosed SLE should start on full-dosage HCQ • Pregnant females with Lupus should remain on the same dose of HCQ • Selected individuals may begin therapy with belimumab [44]
**3.**	If an SLE patient has COVID-19: • Corticosteroids should be maintained to avoid Addisonian episode • HCQ should also be taken to assist prevent illness flare-ups • If an individual feels ill, temporarily stop taking other immunosuppressant drugs
**4.**	SLE conditions necessitate critical clinical requirements such as early warning of a flare-up and vigilant monitoring after vaccination [45].
**5.**	Continuously monitor patients, and give easy access to health care for quick decision making on treatment intensification or de-escalation [46].
**6.**	Avoid drugs, stress and other factors that trigger SLE.

HCQ, hydroxychloroquine; SLE, systemic lupus erythematosus; COVID-19, Coronavirus disease of 2019.

## 2 Conclusion

The current worldwide COVID-19 pandemic and its immunization, have demonstrated several consequences in various individuals, one of which is the development of autoimmune diseases like SLE; many people have been diagnosed with SLE after receiving the mRNA COVID-19 vaccine. Additionally, individuals with underlying SLE have experienced repeated flare-ups of illness after receiving COVID-19 vaccines. The mechanism by which immunization induces SLE is presumably owing to the production of comparable inflammatory mediators in SLE illness and the COVID-19 vaccine. Vigilant monitoring, the use of corticosteroids and a full dosage of hydroxychloroquine (HCQ) are some precautionary measures that can be taken to decrease the risk of SLE following COVID-19 vaccination.

## Sources of funding

None.

## Registration of research studies

1. Name of the registry:
2. Unique Identifying number or registration ID:
3. Hyperlink to your specific registration (must be publicly accessible and will be checked):

## Consent

N/A.

## Guarantor

N/A.

## Provenance and peer review

Not commissioned, externally peer reviewed.

## Funding

None.

## Declaration of competing interest

None.
